# Organoid-based single-cell spatiotemporal gene expression landscape of human embryonic development and hematopoiesis

**DOI:** 10.1038/s41392-023-01455-y

**Published:** 2023-06-02

**Authors:** Yiming Chao, Yang Xiang, Jiashun Xiao, Weizhong Zheng, Mo R. Ebrahimkhani, Can Yang, Angela Ruohao Wu, Pentao Liu, Yuanhua Huang, Ryohichi Sugimura

**Affiliations:** 1 The University of Hong Kong, Hong Kong, China; 2 Centre for Translational Stem Cell Biology, Hong Kong, China; 3 The Hong Kong University of Science and Technology, Hong Kong, China; 4 University of Pittsburgh, Pittsburgh, PA USA

**Keywords:** Stem cells, Differentiation

**Dear Editor**,

The cell-cell interactions are fundamental mechanisms of differentiating embryonic tissues.^1^ We aimed to model human embryonic development in stem-cell-derived organoids. We exploited the time series single-cell and spatial transcriptomics to reveal the gene expression landscape in the development of embryonic tissues and their interactions.

We leveraged the Human EMbryonic Organoid (HEMO) to model the development of embryonic tissues from human pluripotent stem cells proficient in both the extra-embryonic lineage and three germ layers.^2^ HEMO generation in the hematopoietic differentiation condition emulated the process of extra-embryonic lineage and three germ layers before D8, emerging hematopoiesis at D15, and predominant hematopoiesis at D18 (Fig. 1a; Supplementary Fig. 1a; Supplementary Table 1). Among 22,204 cells, we annotated eighteen cell clusters by known marker genes, including human developmental lineages and hematopoiesis (Fig. 1b–d; Supplementary Fig. 1b; Supplementary Table 2). The flow cytometry analysis and immunofluorescence staining confirmed the hematopoietic lineages (Supplementary Fig. 1e, f).

**Fig. 1:**
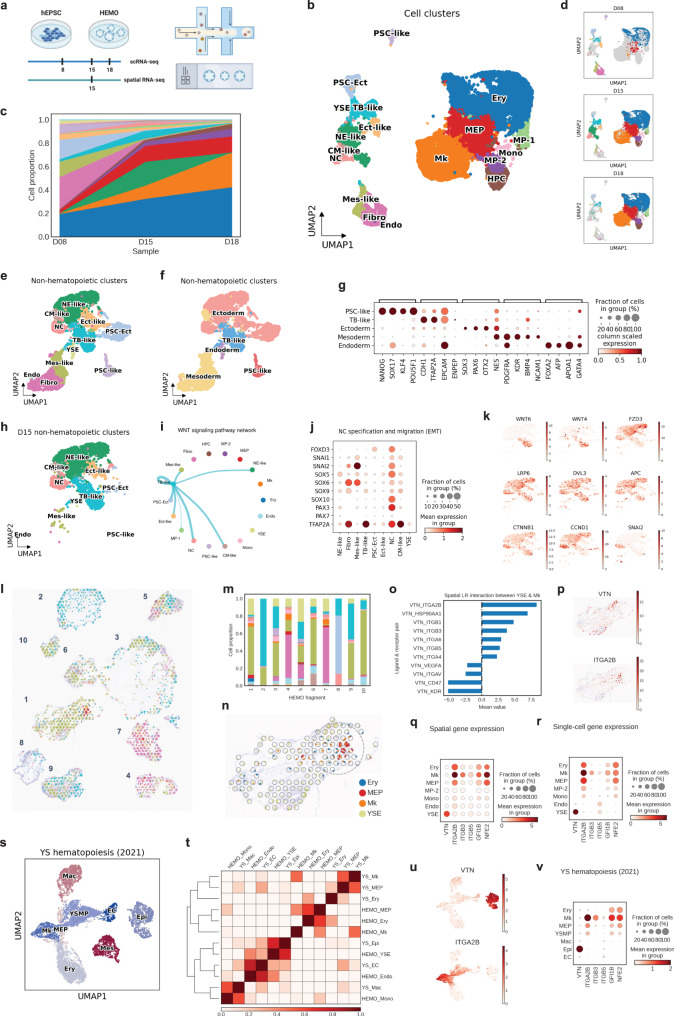
**a** Overall study design. Figure generated by BioRender. Human embryonic organoids (HEMOs) at D8, D15, and D18 were harvested for 10X Chromium scRNA-seq. D15 HEMOs were also harvested for 10X Visium spatial transcriptomics. **b** scRNA-seq analysis of time-series HEMO cells (*n* = 22,204) visualized by UMAP. CM-like cardiac mesoderm-like cells, Ect-like Ectoderm-like cells, Endo endothelium, Ery erythroid cells, Fibro fibroblasts, HPC hematopoietic progenitor cells, MP-1 myeloid progenitor 1, MEP megakaryocyte and erythrocyte progenitors, MP-2 myeloid progenitor 2, Mes-like mesoderm-like cells, Mk megakaryocytes, Mono: monocytes, NC neural crest, NE-like neural ectoderm-like cells, PSC-Ect pluripotent stem cells with ectoderm specification, PSC-like pluripotent stem cells-like, TB-like trophoblast-like cells, YSE yolk sac endoderm. **c** Area chart shows cell proportion changes during the differentiation. **d** Individual UMAP shows cell populations at D8 (*n* = 3124), D15 (*n* = 8350), D18 (*n* = 10,700). **e** UMAP of non-hematopoietic cell clusters. **f** UMAP of non-hematopoietic cell clusters grouped by Immature PSC, TB-like, Ectoderm (PSC-Ect, Ect-like, NE-like, NC), Mesoderm (Mes-like, CM-like, Fibro, Endo) and Endoderm (YSE) populations (*n* = 5477). **g** Marker gene expression of Immature PSC, TB-like, Ectoderm, Mesoderm, Endoderm clusters. **h** UMAP of cell populations in D15 HEMO. **i** WNT signaling pathway interaction among D15 HEMO cell populations. Wider lines indicate stronger interaction. **j** Gene expression pattern of NC specification and migration in D15 non-hematopoietic cells in HEMO. **k** UMAP shows the gene expression of WNT signaling in D15 HEMO. **l** UMAP of cell clusters of decomposed single-cell level spatial transcriptomic data by SpatialScope. **m** Stacked bar plot shows the cell composition of each HEMO. **n** Spatial plot of Ery, Mk, MkP, and YSE population within HEMO #1. The yolk sac erythro-megakaryopoiesis niche is pointed out by a dashed gray circle. **o** Spatial cell-cell interaction analysis conducted by CellPhoneDB. Pairs with a mean value above 0 indicate the activation of the ligand-receptor pairs. Pairs with a mean value below 0 indicate the inhibition of the pairs. Among these, VTN-ITGA2B exhibit the highest enriched score between YSE and Mk. **p** Gene expression pattern of *VTN* and *ITGA2B* on HEMO #1 in a spatial slice. **q** Gene expression of *VTN* and related downstream targets in imputed spatial data in HEMO #1. **r** Gene expression of *VTN* and related downstream targets in scRNA-seq dataset. **t** UMAP analysis of cell clusters of in vivo yolk sac hematopoiesis dataset (Wang et al., 2021). Ery erythrocyte, Mk megakaryocyte, MEP Mk-erythroid progenitor, Mac macrophage, YSMP YS-derived myeloid-biased progenitor, EC endothelial cell, Epi epithelial cell, Mes mesenchymal cell. **s** Heatmap indicates the correlation between HEMO sample and in vivo yolk sac sample. **u** UMAP shows the gene expression of *VTN* and *ITGA2B* in the yolk sac dataset. **v** Dot plot shows the gene expression of *VTN* and related downstream targets in the YS hematopoiesis dataset

To examine three germ-layer patterning of HEMO, we further grouped non-hematopoietic clusters into Immature PSC, TB-like, Ectoderm (PSC-Ect, Ect-like, NE-like, NC), Mesoderm (Mes-like, CM-like, Fibro, Endo) and Endoderm (YSE) (Fig. 1e–g; Supplementary Fig. 2a–c). Extra-embryonic tissues (TB-like, YSE) emerged first, followed by ectoderm and mesoderm (Supplementary Fig. 2d). We noted an overall reduction of non-hematopoietic populations and proportion changes when HEMO predominated hematopoietic fate at D18 (Fig. 1c, d). These observations demonstrate that HEMO followed the development of placenta, endoderm, mesoderm, ectoderm leading to embryonic trunk tissues.

We then investigated the cell-cell interactions during the development of embryonic trunk tissues by CellChat. WNT signaling from TB-like tissues was predominantly received by neural crest populations (Fig. 1h, i; Supplementary Fig. 3a, b). The high expression of neural crest specification genes (*FOXD3, SOX5, SOX6, SOX9, SOX10, PAX3, PAX7, TFAP2A*) and migration genes (*SNAI1, SNAI2)* suggested the maturation of neural crest cells (Fig. 1j; Supplementary Fig. 3c, d). We found that ligands *WNT4* and *WNT6* were expressed from TB-like cells, whilst receptors *LRP6* were expressed in neural crest and neuroectoderm (Fig. 1k). WNT mediators *DVL*, *APC*, and *CTNNB1* lead to the expression of *CCND1* and *SNAI2*, the latter facilitates migration of neural crest. These observations suggest a potential function of TB-like tissues in promoting neural crest maturation and migration in the HEMO.

To examine the cell-cell interactions in hematopoietic tissues, we conducted spatial transcriptomics on HEMOs at D15 when the diversity of tissue types peaked. To gain single-cell level resolution, we jointly analysed the 10X Visium transcriptomes with the matched H&E imaging by SpatialScope, a statistical method which integrates single-cell and spatial transcriptomic data to obtain the spatial distribution of the whole transcriptome at the single-cell resolution. By applying SpatialScope to our datasets, we obtained high resolution gene expression landscape of individual HEMO (Fig. 1l; Supplementary Fig. 4a, b). We observed variability among HEMOs possibly due to different cutting layers and polarization (Fig. 1m; Supplementary Fig. 4c).

We further examined cell-cell interactions in the HEMO #1. YSE was mainly located in the margin of HEMO, while erythroid cells and megakaryocyte lineages appeared nearby YSE. We detected the co-localization of the YSE, Ery and Mk populations in the same spots, suggesting physical cell-cell interactions. Here, we referred to these regions as the yolk sac erythro-megakaryopoietic niche (Fig. 1n, circled; Supplementary Fig. 4e). CellPhoneDB computed the spatial ligand-receptor interaction pairs between YSE, Ery, MkP and Mk. We observed a strong interactive score between YSE and Mk through vitronectin (VTN) signaling, with VTN-ITGA2B ranked highest (Fig. 1o; Supplementary Fig. 5a, b; Supplementary Table 3). Immunofluorescence staining confirmed this niche (Supplementary Fig. 5c). *VTN* was exclusively expressed in YSE, while *ITGA2B* was expressed in the erythro-megakaryopoietic populations, with the highest expression level in Mk (Fig. 1p). Other integrin subtypes *ITGB3* and *ITGB5* as receptors of vitronectin were highly expressed in Mk (Fig. 1q). Notably, *GFI1B* and *NFE2*, involved in ITGB3 signaling, were also expressed in Mk.^3^ Consistently, vitronectin-integrin genes revealed the similar pattern in our paired scRNA-seq dataset and spatial transcriptomics (Fig. 1r). Extracellular matrix regulates megakaryocyte maturation.^4^ ITGA2B inhibition reduced megakaryopoiesis in HEMO (Supplementary Fig. 5d). Although there was variability among HEMOs of different cutting layers, we consistently observed the yolk sac erythro-megakaryopoietic niche in difference spatial slices (Supplementary Figs. 6–9). These findings indicate that yolk sac endodermal cells are the primary source of vitronectin expression, and that the integrin pathway plays a role in promoting megakaryopoiesis in HEMOs.

Finally, we analyzed the scRNA-seq dataset of human fetal tissues (herein called ‘YS hematopoiesis’) in order to identify corresponding tissues to HEMOs.^5^ We identified that fetal yolk sac corresponded with HEMO (Fig. 1s, t). *VTN* was mainly expressed in yolk sac epithelial cells and *ITGA2B* was mainly expressed in megakaryocytes (Fig. 1u). We defined the similar expression pattern of *VTN, ITGA2B, ITGB3, ITGB5, GFI1B*, and *NFE2* between fetal yolk sac and HEMO (Fig. 1v YS hematopoiesis vs Fig. 1r HEMO). HEMOs could mimic human embryonic hematopoiesis in the yolk sac, and vitronectin-integrin signaling as a molecular signature of megakaryopoiesis.

## Supplementary information

Supplementary Material

Supplementary Table 1

Supplementary Table 2

Supplementary Table 3

## Data Availability

The scRNA-seq and spatial transcriptomics data reported in this study have been deposited in NCBI with the accession number PRJNA855311. The link to the dataset. The human yolk sac datasets used in this paper are public available at GSE144024. The scripts used for the analysis of scRNA-seq and spatial transcriptomics have been uploaded to GitHub: https://github.com/CHAOYiming/HEMO_analysis.
