# Supplementary material for: Organoid-based single-cell spatiotemporal gene expression landscape of human embryonic development and hematopoiesis
Source: Signal Transduct Target Ther. 2023 Jun 2;8:230. doi: 10.1038/s41392-023-01455-y (PMC10235070; doi:10.1038/s41392-023-01455-y)
Supplement: Supplementary file 1 — Supplementary Material [file 41392_2023_1455_MOESM1_ESM.docx]

Supplementary Materials for

Organoid-based single-cell spatiotemporal gene expression landscape of human embryonic development and hematopoiesis

Yiming Chao 1,2*, Yang Xiang 1*, Jiashun Xiao 3, Weizhong Zheng 1, Mo R. Ebrahimkhani 4, Can Yang 3, Angela Ruohao Wu 3, Pentao Liu 1,2, Yuanhua Huang 1,2, Ryohichi Sugimura 1,2#

Correspondence to: rios@hku.hk

**This PDF file includes:**

Materials and Methods

Figures. S1 to S9

Tables S1 to S3

Materials and Methods

## HEMO differentiation

**hEPSCs line.** Human M1-hEPSCs were established and kindly gifted by Pentao Liu ^1^.

**hEPSCs maintenance and pre-differentiation.** hEPSCs were maintained and cultured in EPSC medium with medium change every other day. Medium composition was previously reported (Gao et al., 2019) Before EB formation, cells were first pre-differentiated in KSR medium (DMEM/F12 + 10% KSR) (Thermo Scientific, catalog no. 11320033; Thermo Scientific, catalog no. 10828028) for 3 days.

**EB formation in hanging drop.** KSR medium was removed. Cells were washed with PBS (Thermo Scientific, catalog no. 10010023). Cells were further digested with 500mL 0.05% Trypsin (Thermo Scientific, catalog no. 25300054) at 37 °C for 3 min. Trypsin was removed and 2mL KSR medium was added to harvest the cells. Cells were handled gently and avoid harsh pipetting. The collected cells were centrifuged under 300g for 3 min. Supernatant was removed carefully. Add 1mL KSR medium and 1uL Y27632 (Tocris, catalog no. 1254/10) to resuspend the cells and to keep human stem cells viable (Watanabe et al. 2007). 4,000 cells were kept for each 25uL hanging drop on the cap of 10cm petri dish. 30-40 drops were made for each cap. The dish was filled with PBS to keep moist. The cap was gently and slightly inverted to cover the dish. All dishes were kept at 37 °C for 3 days.

**EB collection and differentiation.** All EBs were collected with PBS washing. EBs were further centrifuged under 100g for 1 min with the supernatant removed carefully. The EBs were added with 1mL medium A (STEMdiff Hematopoietic Kit, catalog no. 05310) and transfered to non-adherent 24-well plate (Day 0). HEMOs were cultured in 1mL STEMdiff medium A for 3 days. The medium was half-changed on Day 2. HEMOs were further cultured with 1mL STEMdiff medium B for the following days.

**10x Chromium scRNA-seq sample preparation and sequencing**

**Preparation of single-cell suspensions.** Organoids were harvested at D8, D15, and D18 since EB formation in hanging drop. Organoids were washed with PBS, followed by mechanical chopping with scissors 20-30 times. The tissues were digested in 500μL Accumax (STEMCELL, catalog no. 07921) at 37 °C for 10-15 min and terminated with 500μL PBS with 2% FBS. Cells were collected through the 40μm cell filter. The cell suspension was centrifuged at 500 × g for 5 min and resuspended in FACS sorting buffer (1 × PBS with 2% FBS) (ATCC: The Global Bioresource Center, catalog no. 30-2020) for subsequent staining. Cell concentration was adjusted to around 3,000 cells per μL by counting with a hemocytometer.

**Flow cytometry for scRNA-seq.** Cells were stained in FACS sorting buffer with DAPI (BD Biosciences, catalog no. 564907) in 1:100 for 5 min at 4 °C. Cells were sorted by BD Influx flow cytometry equipment in CPOS at HKUMed. Cells were gated to exclude dead cells and doublets and collected in a chilled single-cell suspension medium (1 × PBS with 0.04% BSA) (Sigma-Aldrich no. A7030) for scRNA-seq library construction. Cell concentration was adjusted to around 500-1000 cells per μL counted with a hemocytometer.

**scRNA-seq library preparation and sequencing.** Single-cell encapsulation, library preparation, and sequencing were done at the University of Hong Kong, LKS Faculty of Medicine, Centre for PanorOmic Sciences (CPOS), Genomics Core. Single-cell encapsulation and cDNA libraries were prepared by Chromium Next GEM Single Cell 3ʹ Reagent Kit v3.1 and Chromium Next GEM Chip G Single Cell Kit. Around 8,000-16,000 live single cells of size 30μm or smaller and of good viability were encapsulated, followed by reverse transcription and library preparation to harvest a pool of cDNA libraries. Libraries were sequenced using Illumina Novaseq 6000 for Pair-End 151bp sequencing. Individual samples had an average throughput of 165.5 Gb.

**Technical replicates and biological replicates.** Each sample of single-cell RNA-seq came from 3-5 technical replicates. 3 biologically independent time points were collected for scRNA-seq.

**10x Visium sample preparation and sequencing**

**Frozen sample preparation.** Fresh HEMOs were collected on Day 15 of differentiation. Washed the HEMOs twice with PBS and transferred the HEMOs into a disposable plastic cryo-mold (Sakura, catalog no. 25608-922), located in the center. Added O.C.T. (Sakura, catalog no. 4583) to immerse the HEMOs fully. Placed the cryo-mold onto the dry ice box until O.C.T. froze (at least 10 min), then stored the cryo-mold immediately at -80C.

**Reagents and slides preparation.** The Visium Spatial Gene Expression Starter Kit (10 x Genomics, catalog no. PN-1000200) was used for the experiment, including the Visium Spatial Tissue Optimization Slide & Reagent Kits (10 x Genomics, catalog no. PN-1000193), Visium Spatial Gene Expression Slide & Reagent Kits (10 x Genomics, catalog no. PN-1000184), and Visium Accessory Kit (10 x Genomics, catalog no. PN-100194).

**RIN (RNA integrity) detection.** Cryosections were cut at 10 µm thickness. Cryosections were flattened out by gently touching the surrounding O.C.T. with cryostat brushes diagonally. At least 25 slices of 10mm cryosections were collected. RNA was extracted and then analyzed by NanoDrop2000 (Thermo Scientific, catalog no. ND2000CLAPTOP). RIN values were acquired from RNA 6000 Nano). The qualified RIN value was over 7.0.

**Tissue Optimization.** The Tissue Optimization kits were used to optimize the tissue permeabilization time. Tissue cryosections were attached, fixed, stained, and permeabilized for different lengths of time. Fluorescent nucleotide was used as an indicator of permeabilization. The permeabilization time was determined by the highest brightness and lowest diffusion of permeabilized samples under the fluorescent microscope. The optimal permeabilization ensured the release of mRNA and minimized the diffusion of mRNA. In this study, tissue permeabilization time ranged from 6 to 12 min.

**Staining and Tissue imaging.** HEMO samples on Day 15 of differentiation were embedded in O.C.T. and snap-frozen by a dry ice box. Cryosections were cut at the 10 µm thickness and attached to the GEX slides. The slides were placed on the Veriti™ 96-Well Fast Thermal Cycler (Applied Biosystems, catalog no. 4375305) and incubated for 1 min at 37C. After incubation, slides were transferred into the pre-chilled methanol at -20C for the fixation, then proceeded to H&E staining. Samples were incubated in isopropanol (catalog no. I9516-25ML) for 1 min, in Hematoxylin (catalog no. MHS16-500ML) for 7 min, after 15 times of quick washing, bluing buffer (catalog no. CS70230-2) for 2 min, and Eosin Mix (catalog no. HT110216-500ML) for 1 min. Slides were incubated onto the thermocycler for 5 min at 37C. The brightfield image was obtained by NanoZoomer (Hamamatsu, catalog no. C13239-01) at 20x resolution.

**cDNA synthesis and Second strand synthesis.** For cDNA synthesis, the stained slides were placed in the Visium Slide Cassette (provided in the Visium kit). The Permeabilization Enzyme (provided in the Visium kit) was added to the wells from the slide cassette. The slide cassette was incubated for 6 min at 37 °C. After washing the wells with 0.1 × SSC (Millipore Sigma, catalog no. S66391L) buffer, we added RT Master Mix (provided in the Visium kit) and initiated reverse transcription for 45 min at 53 °C. After the removal of RT Master Mix, 0.08M KOH (Millipore Sigma, catalog no. P4494-50ML) solutions were added to the well and the slides were incubated for 5 min at room temperature. After washing by Buffer EB (QIAGEN, catalog no. 19086), Second Strand Mix (provided in the Visium kit) was added to each well and the slides were incubated for 15 min at 65 °C. The slides were then washed by Buffer EB, and 0.08M KOH was added to the well for 10 min at room temperature. Samples from each well were collected and transferred to an 8-tube strip containing Tris solution (Thermo Fisher Scientific, catalog no. AM9850G).

**cDNA amplification.** cDNA cycler number was determined by qPCR (Takara Bio, catalog no. RR820A). The Cq Value was determined to be 25% of the peak fluorescence value. With Amp Mix (provided in the Visium kit) and cDNA Primers (provided in the Visium kit), cDNA was amplified with the determined cycle according to the general PCR protocol.

**Spatial Gene Expression Library Construction.** Library construction was carried out with a library construction kit (10 x Genomics, catalog no. PN-1000190) according to the manufacturer’s protocol. cDNA samples were processed by fragmentation, end-repair & A-tailing, SPRI (Solid Phase Reversible Immobilization) selection, adaptor ligation, and index PCR. The libraries were flanked with P5 and P7 sequences.

**Sequencing.** The Visium libraries consisted of standard Illumina constructs flanked with P5/P7. TruSeq Read 1 was used to sequence the spatial barcode and UMI. TruSeq Read 2 was used to sequence the cDNA insert. Sequence depth ranged from 28.4 Gb to 36.3 Gb.

**Technical replicates and biological replicates.** Each sample of 10X Visium came from 3-5 technical replicates.

**Flow cytometry analysis**

Cell suspensions were prepared from HEMO for flow cytometry analysis. Cells were stained in FACS sorting buffer with individual panels. Antibodies for erythroid cells were CD71-PE (BD catalog no. 561938) and CD235a-APC (BD catalog no. 561775). Antibodies for megakaryocytes were CD41a-PE (BD catalog no. 555467) and CD42b-APC (BD catalog no. 551061). Antibody for monocytes was CD16-PeCy7 (BD catalog no. 557744). After antibody staining, cells were stained in with DAPI (BD Biosciences, catalog no. 564907) in 1:100 for 5 min at 4 °C. Cells were sorted by BD Influx flow cytometry equipment in CPOS at HKUMed.

**Immunofluorescence staining and imaging**

D15 HEMO samples were paraffin-embedded and fixed in 4% PFA (Beyotime, catalog no. P0099-500ML) for 1 hour. Paraffin sections were cut at the 3µm thickness and attached to the glass slides. Then the slides were immersed into different solutions (Xylene (Sigma-Aldrich, catalog no. 534056), 100% ethanol, 90% ethanol, 80% ethanol, 70% ethanol, distilled water) separately for 10 min. After hydration, the slides were placed in the recovery solution (Dako, catalog no. S1699-500ML) for 1 hour. The cut slides were permeabilized with 0.5% Triton X-100 (Sigma-Aldrich, catalog no. 93443-100ML) for 30 min and blocked with 10% BSA solution (Millipore Sigma, catalog no. 126615-25ML) for 1 hour. The slides were washed twice and then stained the slides with the primary antibody overnight in a wet box. After twelve hours, slides were rinsed with PBS thrice, then the secondary antibody were added and incubated for one hour. Slides were again rinsed thrice with PBS and 100ul diluted DAPI buffer were added (BD Bioscience, catalog no. 564907) to each slide, and incubated for 3 min at room temperature. Slides were washed with PBS twice and then kept the slides wet. Images were taken by Nikon Ti2E Fluorescent microscope and merged using ImageJ software. Antibody for erythroid cells was anti-CD41 antibody (BD catalog no. 561938). Antibodies for megakaryocytes were anti-CD41 antibody (Abcam catalog no. ab134131) and Goat Anti Rabbit IgG H&L (Abcam catalog no. ab150077). Antibodies for monocytes were anti-CD16 antibody (Abcam catalog no. ab246222), anti-CD68 antibody (Abcam catalog no. ab213363), Goat Anti-Mouse IgG H&L (Abcam catalog no. ab175473) and Goat Anti Rabbit IgG H&L (Abcam catalog no. ab150077). Antibodies for VTN were anti-Vitronectin/S-Protein antibody (Abcam catalog no. ab45139) and Goat Anti Rabbit IgG H&L (Abcam catalog no. ab150077).

**BTT-3033 drug inhibition test**

To functionally test VTN-ITGA2B cellular interaction, BTT-3033 (Purity: 98%) (MedChem Express catalog no. HY-110112) drug was used for *ITGA2B* inhibition. 20μM BTT-3033 was added to the culture medium. Flow cytometry analysis was done to quantify the megakaryocytes proportional changes.

**Data analysis and statistics**

**scRNA-seq data processing and cell filtering.** Sequencing data from 10X Genomics was processed with CellRanger software (version 6.0.0) with GRCh38 human reference genome with default settings, for both reads alignment and initial cell calling. Cell by gene count matrixes was loaded to the Seurat package (version 4.1.0) in R ^2^. Cells with fewer than 1,000 detected genes and with more than 15% mitochondrial gene expression were removed. Genes that were expressed in fewer than 3 cells were also removed. Doublets were detected with the package called DoubletFinder (version 2.0.3) for an individual sample with fine-tuned parameters ^3^.

**Data integration, clustering, and annotation of scRNA-seq data.** After all quality control and doublets removal, the scRNA-seq datasets were integrated with fastMNN (version 1.6.3) ^4^. High variable genes (HVGs) were identified and used for principal component analysis (PCA) for dimension reduction and clustering, all through the Seurat pipeline with default settings. Similarly, markers for each cluster were identified with the FindAllMarkers function and used for cluster annotation. For non-hematopoietic cell clusters, PSC-like, TB-like, PSC-Ect, Ect-like, NE-like, NC, PSC-Ect, Ect-like, NE-like, NC and YSE were re-clustered into different germ-layer populations.

**Cell interaction within scRNA-seq data.** Cell interaction was analyzed by CellChat (version 1.4.0) ^5^. The standard workflow was applied. Gene expression matrix of the individual sample was loaded to CellChat package to calculate the ligand-receptor pair probability. Core functions including computeCommunProb, computeCommunProbPathway and aggregateNet were run following the pipeline. The results were shown in circle graphs.

**Diffusion pseudotime analysis of scRNA-seq data.** The connectivity of the single-cell clusters was first quantified and generated as a partition-based graph abstraction graph (PAGA graph) in Scanpy (version 1.9.1) ^6,7^. For estimating pseudotime, an extended version of diffusion pseudotime (DPT) was applied ^8^. Cells are embedded into force-directed graphs.

**RNA velocity of scRNA-seq data.** Spliced and unspliced RNA were counted by Velocyto (version 0.17.17) and merged with Scanpy adata using loompy function ^9^. RNA velocity was analyzed by UniTVelo (version 0.1.6) ^10^. UniTVelo proposed a unified latent time across the transcriptome, allowing the incorporation of dynamic genes with weak kinetic information. After subsetting D15 ‘HPC’, ‘Mk’, ‘MEP’ cell clusters for megakaryopoiesis and D15 ‘HPC’, ‘MP-1’, ‘MP-2’, ‘Mono’ cell clusters for myelopoiesis, spliced/unspliced information was loaded to UniTVelo for RNA velocity analysis in unified-time mode.

**10x Visium spatial transcriptomics processing.** Sequencing data and imaging tiff files of individual 10x Visium slides were processed with SpaceRanger software (version 1.3.1) with GRCh38 human reference genome. Count matrixes were loaded to Scanpy package (version 4.1.0) in Python. Spots were selected based on RNA counts and mitochondrial gene percentage. After spot selection, HVGs were identified and used for principal component analysis PCA for dimension reduction and clustering. The Scanpy adata objects were used for downstream analysis individually.

**Spatial transcriptomics spot deconvolution.** Two packages were used for spatial transcriptomics spot deconvolution. SpatialScope method was first applied. SpatialScope is a newly developed statistical method which integrates scRNA-seq and spatial transcriptomics data to obtain the spatial distribution of the whole transcriptome at the single-cell resolution (manuscripts in preparation). SpatialScope first performs nuclei segmentation to count and locate nuclei within each spatial spot. Then it assigns a cell type to each located nucleus. Finally, by leveraging the paired scRNA-seq reference data and a learned deep generative model, it decomposes gene expression at each spot into gene expression of the individual cells located within the spot. RCTD package (version 2.0.0) was also used to validate the cell proportion ^11^. It first takes the single-cell reference and predicts the proportion of each cell type in an individual 10x Visium spot. The predicted weighted matrix was used for visualization in the scatter plot (supplementary Fig. 6-9).

**Cell interaction within spatial transcriptomics data.** Two different packages were used for spatial cell-cell interaction analysis. After single-cell resolution spatial data, CellPhoneDB within Squidpy toolbox was first used to predict cellular ligand-receptor interactions ^12,13^. All predicted pairs were first pre-selected by the p-value at 0.001. The enriched score and metadata of individual pairs were referred to. The final results were shown in the bar plot.

**Figure. S1**

Figure. S1. Time-series scRNA-seq of the human embryonic development and hematopoiesis in stem-cell-derived organoids.

**a** Differentiation protocol of human embryonic organoid (HEMO). Human expanded potential stem cells (EPSCs) were maintained in EPSC medium, followed by pre-differentiation and embryonic body (EB) formation in the hanging drop. Experimental design: HEMOs at D8, D15, and D18 were harvested for 10X Chromium scRNA-seq. D15 HEMOs were also harvested for 10X Visium spatial transcriptomics. The right panel illustrates the sample preparation of 10X Chromium scRNA-seq. Figure generated by BioRender. The scale bar = 500 um. **b** Marker gene expression of D8, 15, 18 cell populations. **c** UMAP of D4 HEMO. **d** Marker gene expression of D4 cell populations. **e** Flow cytometry analysis on different hematopoietic populations in HEMO. CD71-PE and CD235a-APC for Ery. CD41a-PE and CD42b-APC for Mk. CD16-PeCy7 for Mono. **f** Immunofluorescence staining on different hematopoietic populations in D15 HEMO. Scale bar = 20um.

**Figure. S2**

Figure. S2. Time-series scRNA-seq of the human embryonic development and hematopoiesis in stem-cell-derived organoids.

**a** UMAP of non-hematopoietic clusters. **b** Identification of cell populations visualized by UMAP. *KLF4*, PSC-like. *CDH1*, TB-like. *OTX2*, ectoderm. *PDGFRA*, mesoderm. *FOXA2*, endoderm. **c** Schematic of early human embryo germ layers, including trophoblast, ectoderm, mesoderm, and endoderm populations. Figure generated by BioRender. **d** Diffusion pseudotime analysis of the developmental trajectory of non-hematopoietic clusters in HEMO. Cells are embedded into force-directed graphs.

**Figure. S3**

Figure. S3. Cellular interactions between TB-like cells and NC populations.

**a** Overall interactome between TB-like cells and NC populations. **b** WNT subtypes between TB-like cells and NC populations. **c** Schematic of WNT signaling and involved genes. EMT, epithelial-mesenchymal transition. Figure generated by BioRender. **d** Circle plots show the signaling pathway network of WNT4 and WNT6 subtypes.

**Figure. S4**

Figure. S4. Spatial transcriptome reveals the yolk sac erythro-megakaryopoietic niche.

**a** Illustration of 10X Visium spatial transcriptomics sample preparation on HEMOs. Figure generated by BioRender. **b** Shannon diversity index of different cell types. A larger number indicates more diverse cell types. **c** UMAP of cell clusters of decomposed single-cell level spatial transcriptomic data by SpatialScope. **d** Scatter plot visualizes cell type deconvolution on HEMO predicted by RCTD. The scale bar indicates the proportion of the specific cell type in each spot. **e** Immunofluorescence staining of yolk sac erythro-megakaryopoietic niche. Scale bar = 20um.

**Figure. S5**

Figure. S5. VTN-ITGA2B interaction within yolk sac erythro-megakaryopoietic niche.

**a** Overall ligand-receptor mapping between YSE and Mk within the yolk sac erythro-megakaryopoietic niche. **b** Selected VTN and integrin-related interactive pairs between YSE and Mk. p-value < 0.001. **c** Immunofluorescence staining of VTN+ YSE cell and CD42b+ megakaryocyte. Scale bar = 20um. **d** Flow cytometry analysis of inhibited integrin α2β1 (ITGA2) with specific inhibitor BTT-3033. **e** Integration of HEMO with human *in vivo* yolk sac dataset (Wang *et al*., 2021).

**Figure. S6**

Figure. S6. HEMO spatial slice 4.

**a** HEMO spatial slice 4 with deconvoluted single cells by SpatialScope.

**Figure. S7**

Figure. S7. HEMO spatial slice 3.

**a** HEMO spatial slice 3 with deconvoluted single cells by SpatialScope.

**Figure. S8**

Figure. S8. HEMO spatial slice 2.

**a** HEMO spatial slice 2 with deconvoluted single cells by SpatialScope.

**Figure. S9**

Figure. S9. HEMO spatial slice 1.

**a** HEMO spatial slice 1 with deconvoluted single cells by SpatialScope.

Table. S1

| Sample information for scRNA-seq and spatial RNA-seq | | | | | |
| --- | --- | --- | --- | --- | --- |
| Sample | Name | Day | Strategy | Cell line | Cell/spot numbers |
| 1 | scrnaseq-d08 | D08 | 10X 3'Chromium | M1-EPSC | 3,124 |
| 2 | scrnaseq-d15 | D15 | 10X 3'Chromium | M1-EPSC | 8,350 |
| 3 | scrnaseq-d18 | D18 | 10X 3'Chromium | M1-EPSC | 10,700 |
| 4 | spatial-d15-a1 | D15 | 10X Visium | M1-EPSC | 417 |
| 5 | spatial-d15-b1 | D15 | 10X Visium | M1-EPSC | 715 |
| 6 | spatial-d15-c1 | D15 | 10X Visium | M1-EPSC | 832 |
| 7 | spatial-d15-d1 | D15 | 10X Visium | M1-EPSC | 1,217 |
| 8 | scrnaseq-d04 | D04 | 10X 3'Chromium | C5-EPSC | 7,292 |

Table. S1. Sample information for scRNA-seq and spatial RNA-seq.

Table. S2.

Table. S2. Top 40 marker gene list for scRNA-seq clusters.

Table. S3.

Table. S3. Spatial ligand-receptor analysis results.

# Supplementary Reference

1. Gao, X. *et al.* Establishment of porcine and human expanded potential stem cells. *Nat Cell Biol* **21**, 687–699 (2019).

2. Hao, Y. *et al.* Integrated analysis of multimodal single-cell data. *Cell* **184**, 3573-3587.e29 (2021).

3. McGinnis, C. S., Murrow, L. M. & Gartner, Z. J. DoubletFinder: Doublet Detection in Single-Cell RNA Sequencing Data Using Artificial Nearest Neighbors. *Cell Syst* **8**, 329-337.e4 (2019).

4. Haghverdi, L., Lun, A. T. L., Morgan, M. D. & Marioni, J. C. Batch effects in single-cell RNA-sequencing data are corrected by matching mutual nearest neighbors. *Nat Biotechnol* **36**, 421–427 (2018).

5. Jin, S. *et al.* Inference and analysis of cell-cell communication using CellChat. *Nat Commun* **12**, 1088 (2021).

6. Wolf, F. A. *et al.* PAGA: graph abstraction reconciles clustering with trajectory inference through a topology preserving map of single cells. *Genome Biol* **20**, 59 (2019).

7. Wolf, F. A., Angerer, P. & Theis, F. J. SCANPY: large-scale single-cell gene expression data analysis. *Genome Biol* **19**, 15 (2018).

8. Haghverdi, L., Büttner, M., Wolf, F. A., Buettner, F. & Theis, F. J. Diffusion pseudotime robustly reconstructs lineage branching. *Nat Methods* **13**, 845–848 (2016).

9. La Manno, G. *et al.* RNA velocity of single cells. *Nature* **560**, 494–498 (2018).

10. Gao, M., Qiao, C. & Huang, Y. UniTVelo: temporally unified RNA velocity reinforces single-cell trajectory inference. 2022.04.27.489808 Preprint at https://doi.org/10.1101/2022.04.27.489808 (2022).

11. Cable, D. M. *et al.* Robust decomposition of cell type mixtures in spatial transcriptomics. *Nat Biotechnol* **40**, 517–526 (2022).

12. Efremova, M., Vento-Tormo, M., Teichmann, S. A. & Vento-Tormo, R. CellPhoneDB: inferring cell-cell communication from combined expression of multi-subunit ligand-receptor complexes. *Nat Protoc* **15**, 1484–1506 (2020).

13. Palla, G. *et al.* Squidpy: a scalable framework for spatial omics analysis. *Nat Methods* **19**, 171–178 (2022).
